# Thanks to all those who reviewed for *Reproductive Health in* 2015

**DOI:** 10.1186/s12978-016-0148-6

**Published:** 2016-03-31

**Authors:** José M. Belizán, Sunni Mumford, João Paulo Souza

**Affiliations:** Institute for Clinical Effectiveness and Health Policy (IECS), Buenos Aires, Argentina; Division of Intramural Population Health Research, of the Eunice Kennedy Shriver National Institute of Child Health and Human Development, National Institutes of Health, Rockville, USA; Intensive Care Unit Department of Obstetrics and Gynecology, School of Medical Sciences, State University of Campinas, Campinas, Brazil

## Abstract

Those devoted to medical science have, as their main purpose, the ambition to improve the quality of life of the population. The editorial team of *Reproductive Health* is mainly devoted to improving reproductive health, particularly for those populations with major needs.

A peer-reviewed journal would not survive without the generous time and insightful comments of the reviewers, whose efforts often go unrecognized. They provide their valuable time without receiving any personal profit to guarantee the quality of research findings. A clear demonstration of generosity, the foundations science was built upon. Final editorial decisions are greatly facilitated by the deeper technical knowledge, scientific insights, understanding of social consequences, and passion that reviewers bring to our deliberations. For these reasons, the Editors-in-Chief and staff of *Reproductive Health* warmly thank the reviewers of 2015 whose comments helped to shape the journal, for their invaluable assistance with review of manuscripts in Volume 12 (2015).

**Erika Aaron**

USA

**Aniekan Abasiattai**

Nigeria

**Nurilign Abebe**

Ethiopia

**Gedefaw Abeje**

Ethiopia

**Saloua Abouchadi**

Morocco

**Roselline Achola**

Uganda

**Ishag Adam**

Sudan

**Abebaw Addis**

Ethiopia

**Temilayo Adeyeye**

USA

**Yohannes Adinew**

Ethiopia

**Jones Adjei**

Canada

**Gabriel Agboado**

UK

**Nadia Ahmad**

United Arab Emirates

**Baldreldeen Ahmed**

Qatar

**Sharon Ahumuza**

Uganda

**David Akeju**

Nigeria

**Moazzam Ali**

Switzerland

**Carmen Alvarez-Nieto**

Spain

**Joshua Amo-Adjei**

Ghana

**Carukshi Arambepola**

Sri Lanka

**Emmanuel Asampong**

Ghana

**José Ayres**

Brazil

**Syed Khurram Azmat**

Belgium

**Syed Khurram Azmat**

Pakistan

**Syed Khurram Azmat**

Canada

**Qadeer Baig**

Pakistan

**Francis Bajunirwe**

Uganda

**Sudharsanam Balasubramaniam**

India

**Justus Kafunjo Barageine**

Uganda

**Dana Barthel**

Germany

**Alka Barua**

India

**Hyam Bashour**

Syrian Arab Republic

**Hyam Bashour**

Syria

**Fereshteh Behmanesh**

Iran

**Regina Benevides**

USA

**Anne-Marie Bergh**

South Africa

**Jane Bertrand**

USA

**Ana Pilar Betran**

Switzerland

**Ana Pilar Betrán**

Switzerland

**Abdeldjellil Bezzaoucha**

Algeria

**Miteshkumar Bhanderi**

India

**Gashaw Biks**

Ethiopia

**Deborah Billings**

USA

**Jesper Bonde**

Denmark

**Ana Luiza Borges**

Brazil

**Andrea Borini**

Italy

**Loretta Brabin**

UK

**Giordana Braga**

Brazil

**Ricardo Cavalli**

Brazil

**Sue Cavill**

UK

**Nomita Chandhiok**

India

**Venkatraman Chandra-Mouli**

Switzerland

**Jeremiah Chikovore**

South Africa

**Antony Chikutsa**

Zimbabwe

**Livia Ciabati**

Brazil

**Jesse Clark**

USA

**John Cleland**

UK

**Kathya Lorena Cordova Pozo**

Bolivia

**Kevin Coughlin**

Canada

**Sienna Craig**

USA

**Doreen Crawford**

UK

**Eugene Kofuor Maafo Darteh**

Ghana

**Dane De Silva**

Canada

**Adriane Delicio**

Brazil

**Zelmen Demeke**

USA

**Markos Desalegn**

USA

**Niveditha Devasenapathy**

India

**Sangappa Dhaded**

India

**Jose Luis Diaz-Rossello**

Uruguay

**Jill Durocher**

USA

**Onyinye Edeh**

Nigeria

**Alison El Ayadi**

USA

**Sebastian Eliason**

Ghana

**Ifeanyichukwu Ezebialu**

Nigeria

**Fabio Facchinetti**

Italy

**Huma Farid**

USA

**Bukola Fawole**

Nigeria

**Gedefaw Fekadu**

Ethiopia

**Janise Ferreira**

Brazil

**Mario Philip Festin**

Switzerland

**Tamara Fetters**

USA

**Tabassum Firoz**

Canada

**Morenike Folayan**

Nigeria

**Florent Fouelifack**

Cameroon

**Ana Franzon**

Brazil

**Ana Galhardo**

Portugal

**Raman Gangakhedkar**

India

**John Ganle**

Ghana

**Osvaldo Ulises Garay**

Argentina

**Abebaw Gebeyehu Worku**

Ethiopia

**Ewenat Gebrehanna**

Ethiopia

**Jillian Gedeon**

Canada

**Pamela Geller**

USA

**Caitlin Gerdts**

USA

**Yoseph Gessesse**

Ethiopia

**Belaineh Girma**

Ethiopia

**Laura Goetzl**

USA

**Jessica Gorman**

USA

**Dereje Habte**

Ethiopia

**Gwyn Hainsworth**

USA

**Partha Haldar**

India

**Kelly Hallman**

USA

**Claudia Hanson**

Sweden

**Karen Hardee**

USA

**Abigail Harrison**

USA

**Emily Harville**

USA

**Abigail Hatcher**

USA

**Amanda Henry**

Australia

**Anna Hjelmstedt**

Sweden

**Johnny Holloway**

USA

**Lianne Holten**

USA

**Christopher Howson**

USA

**Patrick Idoko**

Gambia

**Chibuzo Ilonze**

USA

**Victor Inem**

Nigeria

**Roger Ingham**

UK

**Lindsey Jackson**

USA

**Kapila Jayaratne**

Sri Lanka

**Michael Joffe**

UK

**Nicole Johnson**

USA

**Tanya Jones**

Australia

**Allen Kabagenyi**

Uganda

**Frank Kaharuza**

Uganda

**Alexander Kalimbira**

Malawi

**Shashi Kant**

India

**Helle Karro**

Estonia

**Paul Kawale**

Malawi

**Nancy L Kerr**

USA

**Kavindra Kesari**

Finland

**Assaad Kesrouani**

Lebanon

**Omid Khorram**

USA

**Simon Peter Sebina Kibira**

Uganda

**Caron Kim**

Switzerland

**Theresa Kim**

Canada

**Rolf Klemm**

USA

**Thecla Kohi**

Tanzania

**Yacouba Kone**

Mali

**Theunis Kruger**

South Africa

**Lucy Kululanga**

Malawi

**Manisha Kumar**

India

**Lily C Kumbani**

Malawi

**Amos Laar**

Ghana

**Eve Lackritz**

USA

**Taavi Lai**

Estonia

**Cate Lane**

USA

**Evaline Lang'at**

Kenya

**Margareta Larsson**

Sweden

**Felicia Lester**

USA

**Magdalena Lewicka**

Poland

**Els Leye**

Belgium

**Cheng Li**

China

**Rupeng Li**

USA

**Yan Li**

New Zealand

**Gunilla Lindmark**

Sweden

**Jaime Lopez**

USA

**Beibei Lu**

USA

**Fernando Luiz Affonso Fonseca**

Brazil

**Adriana Gomes Luz**

Brazil

**Moke Magoma**

Tanzania

**Thulani Magwali**

Zimbabwe

**Fabiana Mamede**

Brazil

**Sudharsanam Manni Balasubramaniam**

India

**Abubakar Manu**

Ghana

**Donald Marazzo**

USA

**Mariana Martins**

Portugal

**Magdalena Mattebo**

Sweden

**Susannah Mayhew**

UK

**João Mazzoncini De Azevedo-Marques**

Brazil

**Anthony Mbonye**

Uganda

**Elizabeth Mcclure**

USA

**Sunil Mehra**

India

**Tekleab Mekbib**

Ethiopia

**Tekleab Mekbib**

USA

**Gustavo Mesch**

Israel

**Kara Michels**

USA

**Kristien Michielsen**

Belgium

**Deborah Mindry**

USA

**Suneeta Mittal**

India

**Iryna Mogilevkina**

UKraine

**Lamiya Mohiyiddeen**

UK

**Glen Mola**

Papua New Guinea

**Ilza Monteiro**

Brazil

**Ann Moore**

USA

**Alison Morgan**

Australia

**Olawale Moronkola**

Nigeria

**Leonardo Moscovici**

Brazil

**Kidza Mugerwa**

Uganda

**Stembile Mugore**

USA

**Wilson Muhwezi**

Uganda

**Saiqa Mullick**

South Africa

**Erik Munroe**

UK

**Fabien Munyaneza**

Rwanda

**Jane Namasasu**

Malawi

**Nehemiah Nando**

Zimbabwe

**Enock Ngome**

Botswana

**Jm Nicholson**

USA

**Dabere Nigatu**

Ethiopia

**Nik Daliana Nik Farid**

Malaysia

**Carolyne Njue**

Kenya

**Vicky Nogueira Pileggi**

Brazil

**Vicky Nogueira-Pileggi**

Brazil

**Linda Nyondo**

Malawi

**Francis Obare**

Kenya

**Mary Obiyan**

Nigeria

**Jeanne O'Brien**

USA

**Clifford Odimegwu**

South Africa

**Babasola Okusanya**

UK

**Funmilola Olaolorun**

USA

**Berend Olivier**

Netherlands

**Lemessa Oljira**

Ethiopia

**Comfort Olorunsaiye**

USA

**Karen Pak Oppenheimer**

USA

**Ngozi Orazulike**

Nigeria

**Katrien Oude Rengerink**

Netherlands

**Tia Palermo**

Italy

**Louis Peeters**

Netherlands

**Pascal Petitet**

France

**Sharon Phillips**

USA

**Cynthia Pileggi-Castro**

Brazil

**Torie Plowden**

USA

**Zahida Qureshi**

Kenya

**Sibylle Rahlenbeck**

Germany

**Laila Rahman**

Canada

**Vibeke Rasch**

Denmark

**Krishna Regmi**

UK

**Tamar Renaud**

USA

**Jennifer Requejo**

Switzerland

**Heidi Reynolds**

USA

**Atif Riaz**

Pakistan

**Liesbeth Rijsdijk**

Netherlands

**Michael Ripple**

USA

**Narjis Rizvi**

Pakistan

**Nuriya Robinson**

USA

**Haleh Sangi-Haghpeykar**

USA

**Diane Santa Maria**

USA

**Belen Sanz Barbero**

Spain

**Katherine Sapra**

USA

**Karen Schliep**

USA

**Agumasie Semahegn**

Ethiopia

**H Senanayake**

Sri Lanka

**Carrie Shandra**

USA

**Gaurav Sharma**

UK

**Sumedha Sharma**

Canada

**Dorothy Shaw**

Canada

**Muhammad Ishaque Sheikh**

Pakistan

**Sana Sheikh**

Pakistan

**Chander Shekhar**

India

**Sonjelle Shilton**

USA

**Estelle Monique Sidze**

Kenya

**Robert Silver**

USA

**Maria Asuncion Silvestre**

Philippines

**Sara Simonsen**

USA

**Joao Souza**

Brazil

**Joao Paulo Souza**

Brazil

**Joao Paulo Souza**

Switzerland

**Ilene Speizer**

USA

**Chandrashekhar Sreeramareddy**

Nepal

**Joseph Stanford**

USA

**Rachel Steward**

USA

**Elizabeth Stringer**

USA

**Joar Svanemyr**

Norway

**Gunilla Sydsjo**

UK

**Bee Kang Tan**

UK

**Diane Taylor**

USA

**Danielle Teixeira**

Brazil

**Tesfalidet Tekelab**

Ethiopia

**Zelalem Teklemariam**

Ethiopia

**Marleen Temmerman**

Switzerland

**Britt Pinkowsky Tersbøl**

Denmark

**Gezahegn Tesfaye**

Ethiopia

**Stefanie Theuring**

Germany

**Myrte J Tielemans**

Netherlands

**Tizta Tilahun**

Ethiopia

**Aytekin Tokmak**

Turkey

**Sonila Tomini**

Netherlands

**Nnanna Ugwu**

Nigeria

**Vivian UKah**

Canada

**Priti Upadhyay**

Nepal

**Marcelo Urquia**

Canada

**Anke Van Der Kwaak**

Netherlands

**Carolina Vieira**

Brazil

**Elisabeth Vieira**

Brazil

**Joshua Vogel**

Switzerland

**Elisa Wells**

USA

**Paige Williams**

USA

**Deborah Wing**

USA

**Eskinder Wolka**

Ethiopia

**Eileen Yam**

USA

**Kai Yao**

USA

**Tebikew Yeneabat**

Ethiopia

**Engida Yisma**

Ethiopia

**Nina Zamberlin**

Argentina

**Xiao-Ming Zhu**

China

